# Irradiation-Induced Up-Regulation of HLA-E on Macrovascular Endothelial Cells Confers Protection against Killing by Activated Natural Killer Cells

**DOI:** 10.1371/journal.pone.0015339

**Published:** 2010-12-16

**Authors:** Isabelle Riederer, Wolfgang Sievert, Günther Eissner, Michael Molls, Gabriele Multhoff

**Affiliations:** 1 Department of Radiation Oncology, Klinikum rechts der Isar, Technische Universität München, Munich, Germany; 2 Clinical Cooperation Group (CCG) “Innate Immunity in Tumor Biology”, Helmholtz Zentrum München, Munich, Germany; 3 Department of Cardiac Surgery, Klinikum der Universität München, Munich, Germany; Dresden University of Technology, Germany

## Abstract

**Background:**

Apart from the platelet/endothelial cell adhesion molecule 1 (PECAM-1, CD31), endoglin (CD105) and a positive factor VIII-related antigen staining, human primary and immortalized macro- and microvascular endothelial cells (ECs) differ in their cell surface expression of activating and inhibitory ligands for natural killer (NK) cells. Here we comparatively study the effects of irradiation on the phenotype of ECs and their interaction with resting and activated NK cells.

**Methodology/Principal Findings:**

Primary macrovascular human umbilical vein endothelial cells (HUVECs) only express UL16 binding protein 2 (ULBP2) and the major histocompatibility complex (MHC) class I chain-related protein MIC-A (MIC-A) as activating signals for NK cells, whereas the corresponding immortalized EA.hy926 EC cell line additionally present ULBP3, membrane heat shock protein 70 (Hsp70), intercellular adhesion molecule ICAM-1 (CD54) and HLA-E. Apart from MIC-B, the immortalized human microvascular endothelial cell line HMEC, resembles the phenotype of EA.hy926. Surprisingly, primary HUVECs are more sensitive to Hsp70 peptide (TKD) plus IL-2 (TKD/IL-2)-activated NK cells than their immortalized EC counterpatrs. This finding is most likely due to the absence of the inhibitory ligand HLA-E, since the activating ligands are shared among the ECs. The co-culture of HUVECs with activated NK cells induces ICAM-1 (CD54) and HLA-E expression on the former which drops to the initial low levels (below 5%) when NK cells are removed. Sublethal irradiation of HUVECs induces similar but less pronounced effects on HUVECs. Along with these findings, irradiation also induces HLA-E expression on macrovascular ECs and this correlates with an increased resistance to killing by activated NK cells. Irradiation had no effect on HLA-E expression on microvascular ECs and the sensitivity of these cells to NK cells remained unaffected.

**Conclusion/Significance:**

These data emphasize that an irradiation-induced, transient up-regulation of HLA-E on macrovascular ECs might confer protection against NK cell-mediated vascular injury.

## Introduction

The endothelial cell (EC) monolayer which lines blood vessels performs multiple tasks including the regulation of tissue fluid homeostasis and blood cell transmigration. The interaction of ECs with leukocytes, such as activated NK cells, involves attachment and rolling which are predominantly mediated by selectins and carbohydrate-rich ligands, such as mucins [1]–[3]. Lymphocyte rolling might induce activation and facilitate the tight adhesion to ECs which is enabled by the integrin family of cell adhesion molecules [4]. The ligation, migration and extravasation of lymphocytes into the tissue has been postulated to be mediated by PECAM-1 (CD31) and involves the activation of integrins such as α4/β1 (VLA-4) [4], [5], α4/β7 [5], [6], α4/β2 (LFA-1, CD11a/CD18) [5]–[7], and αM/β2 (CR3, Mac-1, CD11b/CD18a) [7].

NK cells play pivotal roles in the early defence against viral infections and malignant transformations [8]–[10]. The functionality of NK cells is regulated by interactions of inhibitory and activating receptors [11], [12] which belong either to the immunoglobulin (Ig)-like (KIR), Ig-like transcript (ILT), C-type lectin or natural cytotoxicity group, with the signals being derived from the target cell ligands [13]. Inhibitory receptors with specificity for classical and non-classical MHC class I molecules mediate protection for the target cells. HLA-E, a prominent member of the non-classical MHC (MHC class Ib), which is recognized by CD94/NKG2A (inhibitory) and CD94/NKG2C (activating) expressing NK cells, is characterized by a limited polymorphism and a tissue-specific and inducible expression pattern [14], [15]. According to the “missing-self” theory [16], the partial or complete loss of MHC antigens can also render target cells susceptible to NK cells. The mechanism of killing by NK cells involves exocytosis of cytolytic granules containing apoptotic enzymes, such as granzymes and perforins and death receptor ligands such as Fas/Fas-ligand [17]–[24]. Our group has identified a membrane form Hsp70 on tumor cells [25] as a tumor-specific recognition structure for a perforin-independent, granzyme B-mediated attack by allogeneic and autologous NK cells that have been pre-stimulated with Hsp70 peptide TKD plus low dose IL-2 [26], [27].

Activated NK cells have been found to bind to the microvasculature of metastases [28] and to play a crucial role in the control of tumor angiogenesis [29], [30]. In this context, we were interested to study the interactions of TKD/IL-2- stimulated NK cells with macro- and microvascular primary and immortalized ECs. An NK cell based phase I clinical study [31] which used *ex vivo* TKD/IL-2-activated NK cells to treat patients with colorectal and non-small cell lung (NSCLC) carcinoma has shown promising results with respect to the feasibility and safety of the procedure and its ability to enhance the capacity of patient-derived NK cells to kill membrane Hsp70^+^ tumor cells *in vitro*. The goal of a subsequent proof-of-concept phase II clinical trial is to analyze the efficacy of TKD/IL-2-activated NK cells in NSCLC patients following radiochemotherapy as this has been shown to enhance the density of Hsp70 membrane expression on tumor cells (unpublished observations). Therefore, we were interested to study the surface expression of activating and inhibitory ligands on primary and immortalized macro- and microvascular ECs before and after irradiation.

Immortalized EC lines present Hsp70 on their plasma membrane and thus reflect a tumorigenic phenotype. The expression of ligands such as ULBP1-3 [32], MIC-A/-B, Hsp70, and HLA-E and the membrane-bound [33] form of the intercellular adhesion molecule ICAM-1 (CD54) was then correlated with the NK cell mediated cytotoxicity. Our findings suggest that HLA-E is a key player in the regulation of NK cell mediated killing of ECs. A movie showing the interaction of TKD/IL-2-activated NK cells and ECs indicates that a bulk of NK cells actively migrates towards ECs. Next, we studied the effects of ionizing irradiation on the expression of cell surface markers which might affect the killing activity of NK cells. We could demonstrate that an irradiation at 4 Gy results in a significantly up-regulated expression density of HLA-E on primary (HUVEC) and immortalized macrosvascular (EA.hy926) ECs, but not on immortalized microvascular HMEC. Despite a minor irradiation-induced increase in activating ligands, the significant increase in the HLA-E expression on macrovascular ECs led to a reduced lysis of HUVECs and EA.hy926 cells. The HLA-E expression density on the immortalized microvascular cell line HMEC did not change significantly by irradiation and thus the lysis remained unaltered.

## Results

### Comparative Analysis of the Phenotype of Human Primary and Immortalized Macro- and Microvascular ECs

**Figure 1:**
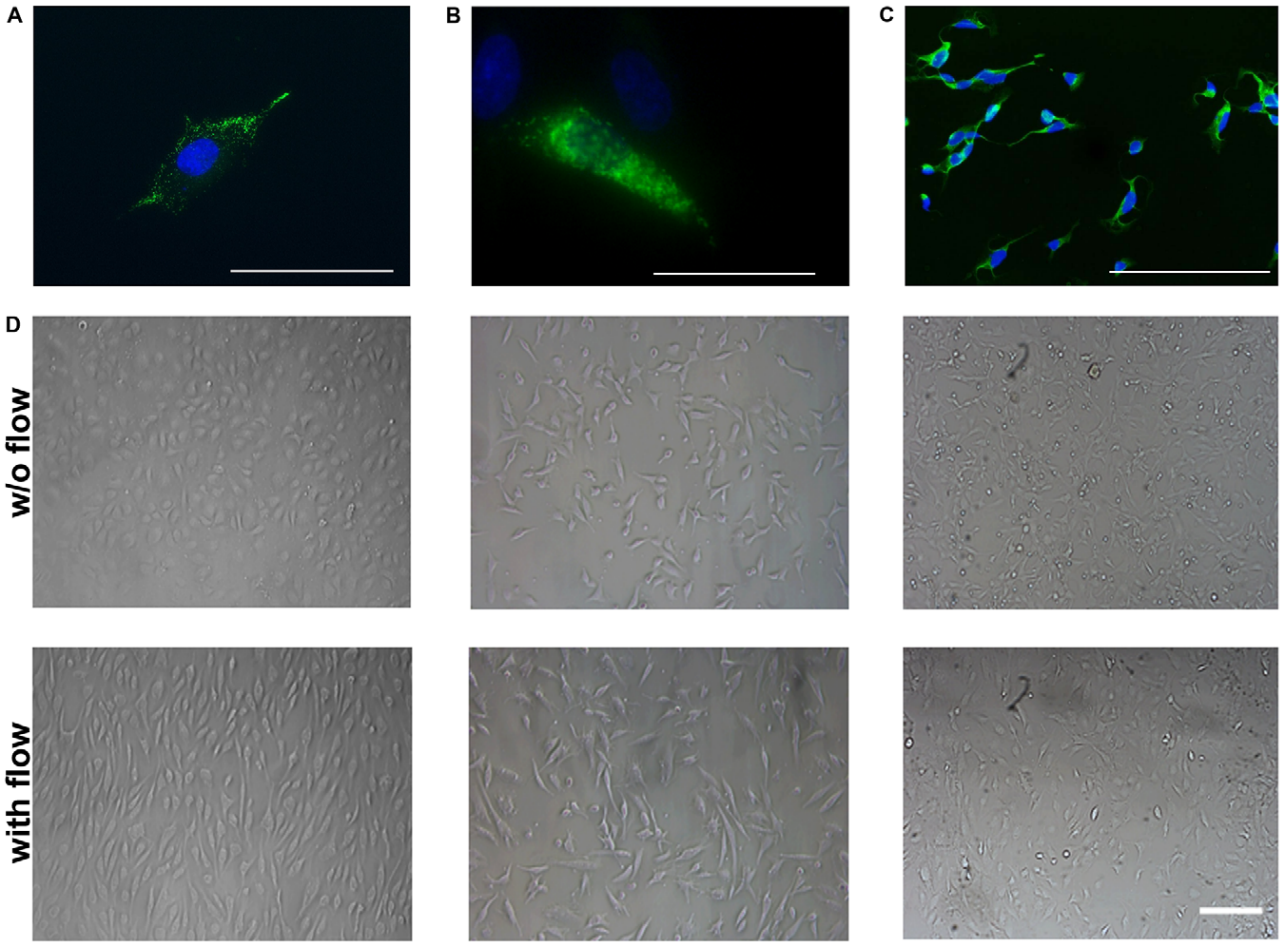
Von Willebrand staining and microscopic analysis of endothelial cells (ECs). Representative immunofluorescence images of primary macrovascular HUVECs (A), immortalized macrovascular EA.hy926 cells (B) and immortalized microvascular HMECs (C) stained for von Willebrand factor (FITC, green spectrum); nucleus is counter-stained with DAPI (blue spectrum); scale bar, 100 µm. (D) Microscopic analysis of HUVECs (left panel), EA.hy926 cells (middle panel) and HMECs (right panel) under static (upper panel) and under flow conditions for 12 h (lower panel), scale bar, 200 µm. Under flow the ECs show the typical cobblestone morphology.

The phenotype of the primary (HUVEC; Figure 1A) [34] and immortalized macro- (EA.hy926; Figure 1B) and immortalized microvascular (HMEC; Figure 1C) endothelial cells was determined by immunofluorescence microscopy and flow cytometry. All three endothelial cell types show a positive staining for the factor VIII-related antigen (Figure 1A–C) and strongly express the endothelial cell associated markers CD31 (platelet/endothelial cell adhesion molecule 1, PECAM-1), [35] and CD105 (endoglin) [36], a component of the TGF-beta 1 receptor complex, which is also involved in vascular remodelling (Table 1). Under shear stress induced by a flow system (IBIDI, Martinsried, Germany) for 12 h (Figure 1D; lower panel), but not under static culture conditions (Figure 1D; upper panel), HUVECs (Figure 1D, left), EA.hy926 cells (Figure 1D, middle) cells and to a lesser extent also HMECs (Figure 1D, right) exhibit the typical cobblestone morphology and a reorientation in line with the dynamic flow.

**Table 1:** Comparative analysis of the expression of activating and inhibitory cell surface markers on primary and immortalized ECs.

	Primarymacrovascular	Immortalized	Immortalizedmicrovascular	ExpressionPattern
ECs	HUVEC	EA.hy926	HMEC	HUVEC/EA.hy926/HMEC
Marker	% (mean fluorescence intensity)			++ >70%+ >20%− >10%
CD31	89±4 (89)	96±7 (96)	97± (92)	++/++/++
CD105	97±6 (97)	99±0.1 (99)	99±2 (98)	++/++/++
ULBP1	0±1 (66)	0±0 (46)	4±1 (43)	−/−/−
ULBP2	48±7 (59)	96±2 (63)	98±1 (131)	+/++/++
ULBP3	6±3 (52)	58±11 (52)	51±11 (62)	−/+/+
MIC-A	73±6 (107)	21±4 (46)	8±3 (116)	++/+/−
MIC-B	3±1 (47)	2±1 (25)	36±10 (37)	−/−/+
HLA-E	1±0 (11)	86±3 (162)	83±4 (201)	−/++/++
W6/32	97±2 (48)	98±9 (75)	95±7 (67)	++/++/++
cmHsp70.1	6±4 (12)	58±5 (69)	48±6 (54)	−/+/+
SPA810	2± (51)	1± (91)	2± (44)	−/−/−
CD54	5±2 (88)	13±2 (46)	21±3 (49)	−/+/+

In contrast to the immortalized ECs, the primary macrovascular human umbilical vein endothelial cells (HUVECs) only present UL16 binding protein 2 (ULBP2), the major histocompatibility complex (MHC) class I chain-related protein MIC-A and classical MHC class I antigens (HLA-A, -B, -C) on their cell surface. A comparative analysis of the expression pattern of MIC-A, ICAM-1 (CD54) and HLA-E expression on ECs under static and flow conditions revealed no significant differences (data not shown).

In comparison to HUVECs, the immortalized EA.hy926 cell line expresses additional markers such as ULBP3, ICAM-1 (CD54) and the human leukocyte antigen E (HLA-E) on their cell membrane. The human microvascular endothelial cell line HMEC was immortalized by a transfection of primary dermal ECs with a PBR322 plasmid containing the coding region for the simian virus 40A gene product, large T antigen. This cell line resembles the phenotype of EA.hy926 cells with the exception that the MIC-A expression is substituted by MIC-B in HMECs. Although a translocation of the major stress-inducible heat shock protein 70 (Hsp70) to the plasma membrane [36], [37] is typically associated with a tumorigenic phenotype, since normal cells are membrane Hsp70^−^, a membrane Hsp70^+^ phenotype was selectively detectable on immortalized EA.hy926 cells and HMECs using the cmHsp70.1 mAb (multimmune GmbH, Munich, Germany), but not on primary HUVECs. In contrast to the cmHsp70.1 mAb, other commercially available Hsp70-specific antibodies, such as SPA810, do not react with the membrane form of Hsp70 on tumor cells (manuscript submitted) or that on immortalized ECs.

### Interaction of EC with Un-Stimulated and Stimulated NK Cells

**Figure 2:**
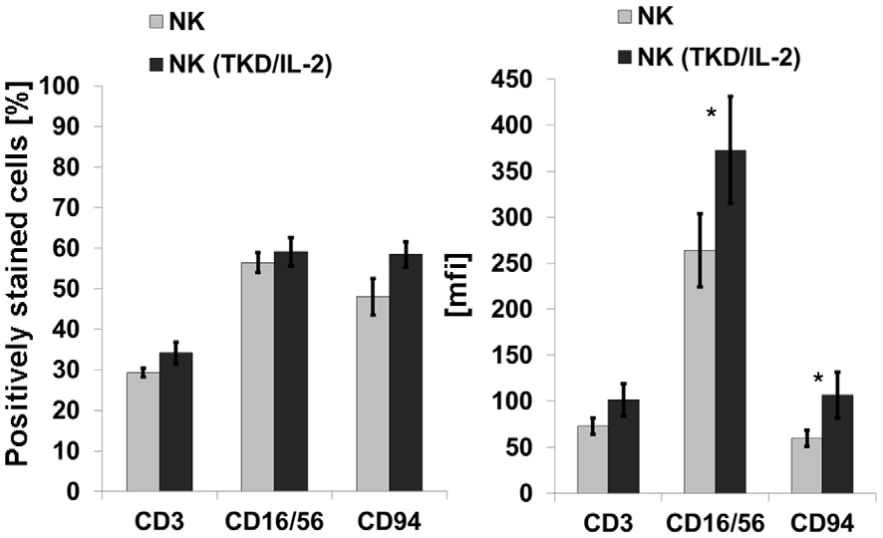
Phenotyping of effector cells. Comparative analysis of T (CD3) and NK (CD16/56, CD94) cell specific markers on NK cell enriched PBMNC preparations with and without stimulation with TKD (2 µg/ml) plus low dose IL-2 (100 IU/ml) for 4 days. Left panel, proportion of marker positively stained cells in %; right panel, mean fluorescence intensity (mfi) values. Mean fluorescence values differing significantly between un-stimulated and stimulated NK cells are marked with *, p<0.05.

The enrichment of NK cells was based on a negative selection procedure of peripheral blood mononuclear cells (PBMNCs) using Miltenyi microbeads coupled to anti-CD3 (T cell depletion) and anti-CD19 (B cell depletion) mAbs. Following the negative selection, an adherence step was included to reduce the number of CD14^+^ monocytes and macrophages. The final population typically contained between 50 and 60% of CD3^−^ NK cells, which were found to be positive for the neuronal cell adhesion molecule CD56 [38], the low affinity Fc-gamma receptor CD16 and the C-type lectin receptor CD94 (Figure 2). Enriched NK cells were either un-stimulated (Figure 2, grey bars) or were stimulated with Hsp70 peptide TKD plus low dose IL-2 (TKD/IL-2) for 4 days (Figure 2, black bars). Although no significant changes were observed with respect to the percentage of CD16, CD56 and CD94 positive cells, the stimulation with TKD/IL-2 resulted in a significant up-regulation in the expression density (mfi) of these antigens (Figure 2).

**Figure 3:**
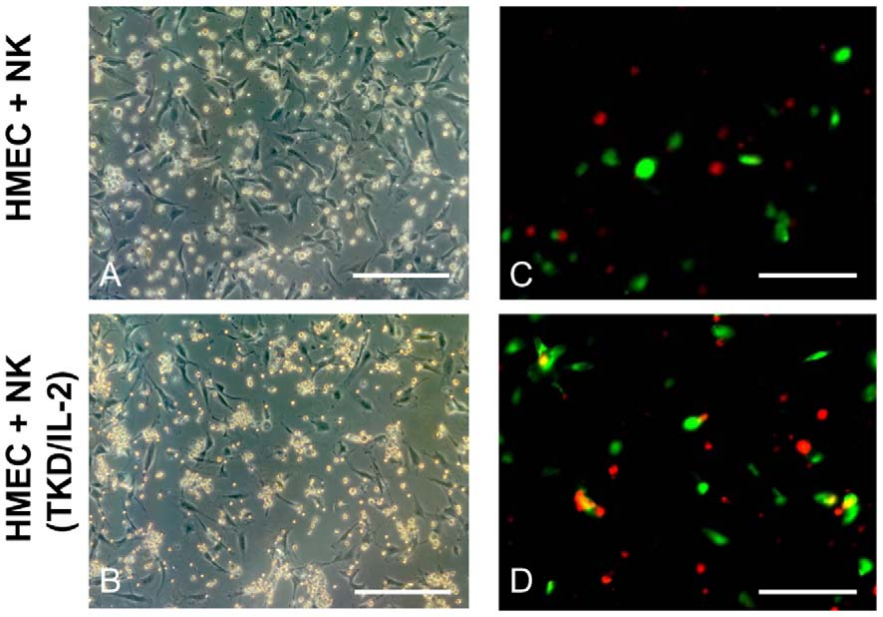
Representative microscopic images of the adherence of TKD/IL-2-activated NK cells to HMECs. A confluent monolayer of HMECs was co-cultured for 4 h either with resting (A) and TKD/IL-2-activated NK cells (B). Pictures were taken using a Zeiss Axiovert 200 inverted fluorescence microscope. The amount of adherent HMECs was drastically reduced after a co-incubation with activated (B) compared to resting NK cells (A). The number of HMECs co-cultured for 4 h with activated NK cells significantly dropped from 73±5 to 37±3 cells, as determined by counting of an optical field (n = 3, p<0.05). Scale bar, 50 µm. Representative immunofluorescence images of TKD/IL-2-activated NK cells to HMECs. The specific adhesion of activated NK cells, labeled with PKH26GL in red, to HMECs, labeled with FITC in green, is illustrated in orange (D). No co-staining of red and green labels was seen when resting NK cells were used (C). Pictures were taken using a Zeiss Axiovert 200 inverted fluorescence microscope.

Differences in the adhesion of un-stimulated and TKD/IL-2-activated NK cells to HMECs under static conditions using microscopic and immunofluorescence analysis of PKH26GL-labeled NK cells (red fluorescence) and FITC-labeled (green fluorescence) HMECs are illustrated in Figure 3. Following a 4 h co-incubation, the number of ECs in culture wells containing activated NK cells (Figure 3B) was significantly reduced from 73±5 to 37±3 ECs per optical field (p<0.05; n = 3), compared to culture wells containing resting NK cells (Figure 3A). Furthermore, the adhesion of activated NK cells to ECs was dramatically greater than that to resting NK cells. The close interaction of activated NK cells with ECs was confirmed using fluorescence microscopy. The adherence of activated PKH26GL-labeled NK cells (red) to FITC-labeled HMECs (green) was visualized in an orange spectrum (Figure 3D). In contrast, no co-localization was detectable if HMECs were co-cultured for 4 h with resting NK cells (Figure 3C). Movies illustrating the differences in the kinetics of the adherence and the concerted attack of HMECs by TKD/IL-2-stimulated NK cells in comparison to resting NK cells are included in the supplementary information. It appears that activated (video S2 and video S3), but not resting NK cells (video S1), actively migrate towards HMECs and that this interaction results in the detachment and elimination of HMECs.

### HLA-E Expression Partially Protects EA.hy926 Cells and HMECs from Lysis by Activated NK Cells

**Figure 4:**
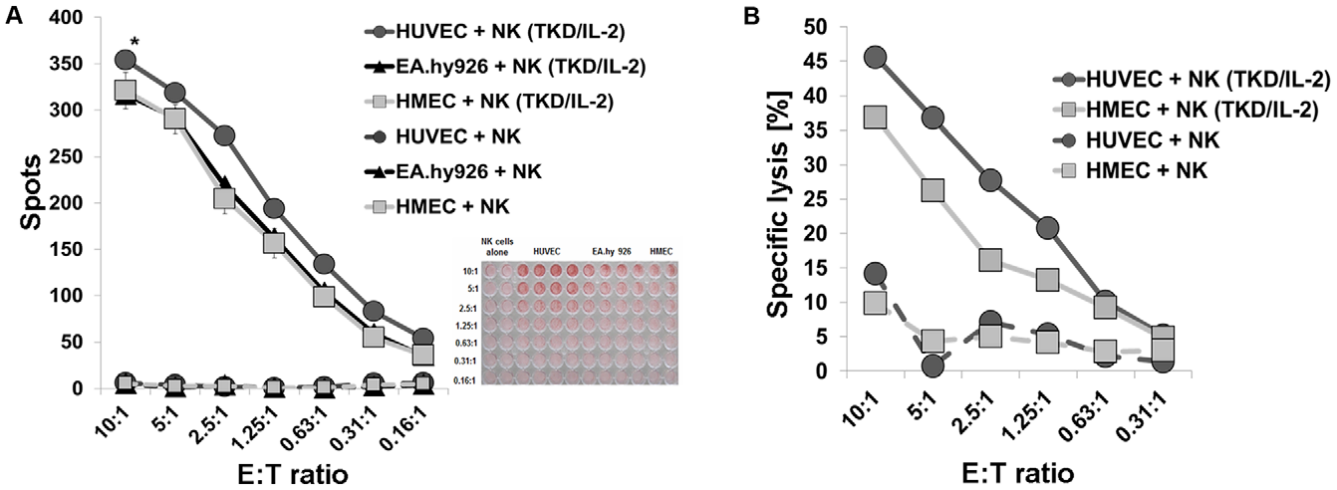
Granzyme B and ^51^Cr release assay of ECs by resting and activated NK cells. Granzyme B ELISPOT assay (4 h) of resting and TKD/IL-2-activated NK cells attacking HUVECs, EA.hy926 cells and HMECs (A). The effector to target (E∶T) ratios ranged between 10∶1 to 0.16∶1. The data represent mean values of at least 3 independent experiments. The differences in lysis of HUVECs compared to that of EA.hy926 cells and HMECs was significantly different *, p<0.05 at all E∶T ratios. Standard ^51^Cr release assay (4 h) comparing the lysis of HUVECs and HMECs by resting and TKD/IL-2-activated NK cells at effector to target ratios ranging from 10∶1 to 0.31∶1 (B). The data are from one typical experiment, therefore no statistical analysis has been performed.

We have previously shown that TKD/IL-2-activated NK cells kill their tumor target cells via a perforin-independent, granzyme B mediated lysis [27]. HUVECs, EA.hy926 cells and HMECs were sensitive to granzyme B mediated apoptosis by TKD/IL-2-activated NK cells (Figure 4A), whereas none of the three ECs were lysed to a significant extent by resting NK cells (data not shown). However, the lysis of primary macrovascular HUVECs by activated NK cells was always considerably greater than that of its corresponding immortalized cell line EA.hy926 and the immortalized microvascular cell line HMEC (Figure 4A/B). This finding was not expected, since the immortalized ECs express ULBP3 and membrane Hsp70, in addition to ULBP1 and MIC-A/-B as activating ligands for TKD/IL-2-stimulated NK cells. We therefore assumed that the increased susceptibility of HUVECs to activated NK cells was most likely due to the absence of the inhibitory ligand HLA-E. A comparison of the lysis of HUVECs and HMECs in a standard ^51^Cr release assay confirmed the data that were obtained using the granzyme B ELISPOT assay (Figure 4B).

### Phenotypic Changes of Cell Surface Markers on ECs after Contact with Activated NK Cells

**Figure 5:**
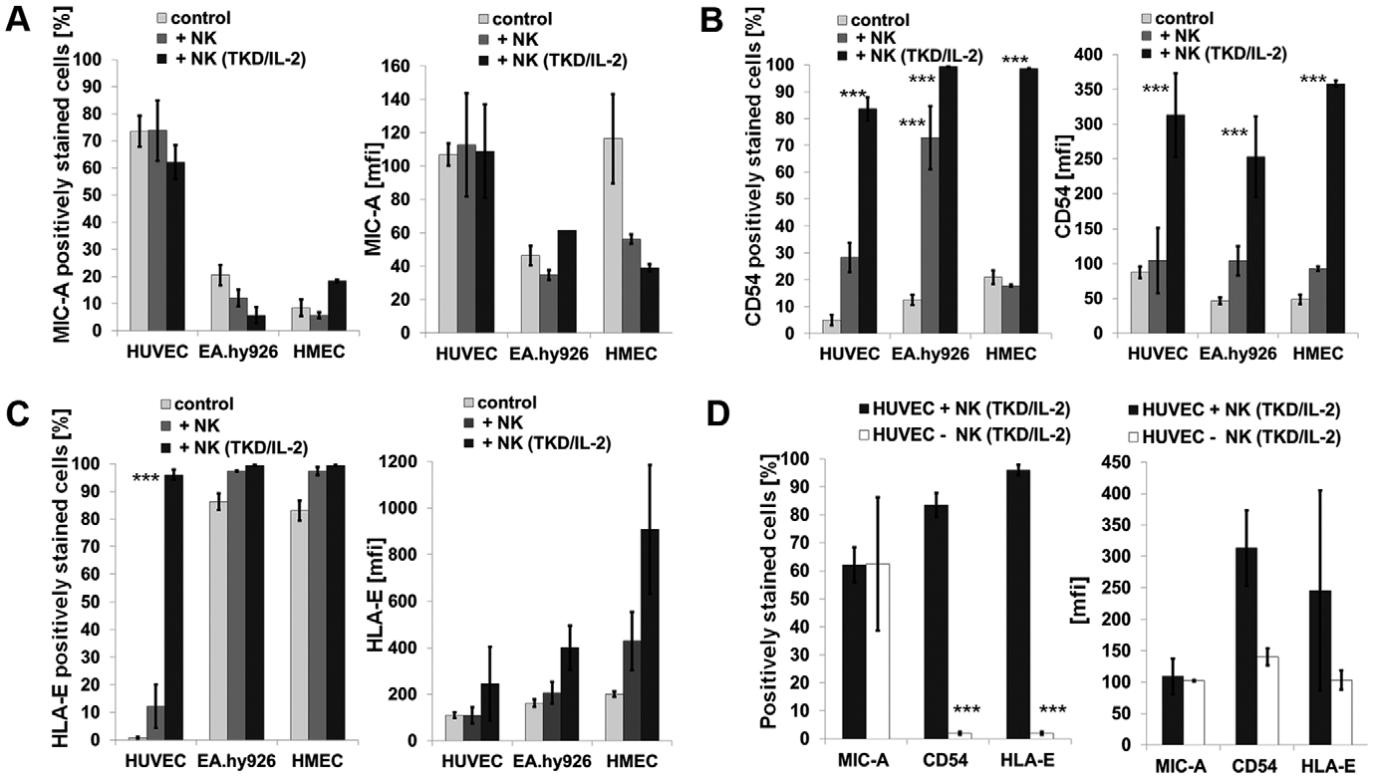
Phenotyping of ECs after contact with resting and activated NK cells. Comparative analysis of the proportion of HUVECs, EA.hy926 cells and HMECs expressing MIC-A (A), CD54 (B) and HLA-E (C) (left panel) and mean fluorescence intensity values of expression (mfi; right panel) in the absence of NK cells (light grey bars) and after a 12 h co-culture with resting (dark grey bars) or TKD/IL-2-activated (black bars) NK cells. Cell surface marker expression was determined by flow cytometry; ECs and NK cell populations were gated separately based on differences in the forward (FSC) and side scatters (SSC). Asterisks mark values significantly different to control values, ***, p<0.001, as determined by the Mann-Whitney test using the SPSS software. The expression of the cell surface markers indicated above on HUVECs in the presence of TKD/IL-2-activated NK cells (black bars) and after removal of the NK cells (white bars) was determined by flow cytometry. In the absence of NK cells the elevated levels in the expression of CD54 and HLA-E positively stained HUVECs dropped to initially low levels (D). Asterisks mark values which are significantly different to control values, ***, p<0.001, as determined by the Mann-Whitney test using the SPSS software.

The proportion of marker positive target cells and the cell surface density of the markers following co-incubation of the ECs with resting and activated NK cells for 12 h were analyzed by flow cytometry in the separately gated cell populations. Apart from MIC-A, ICAM-1 (CD54) and HLA-E, none of the cell surface markers appeared to be affected by the contact with NK cells. The cell surface density of CD31 and CD105, as typical EC-related markers, also remained unchanged (data not shown). As summarized in Figure 5A (left panel), the percentage of MIC-A^+^ primary HUVECs slightly dropped, as did the proportion of MIC-A^+^ in its corresponding immortalized partner cell line EA.hy926. The cell surface density of MIC-A on the surviving cell fraction remained unaltered in HUVECs and EA.hy926 cells (Figure 5A, right panel). The microvascular cell line HMEC, which contains a very low proportion of MIC-A^+^ cells showed a decrease in the surface density of this marker (Figure 5A, right panel), and there was also a small reduction in the proportion of MIC-B^+^ cells (data not shown). With respect to the intercellular adhesion molecule ICAM-1 (CD54), all three EC types reacted similarly by significantly up-regulating the percentage of cells expressing this adhesion molecule and its cell surface density following contact with activated NK cells (Figure 5B). The most striking difference between the EC types was observed with respect to the expression of HLA-E. After contact with activated NK cells, but not with resting NK cells, the percentage of HLA-E^+^ HUVECs increased from below 5% up to nearly 100% within 12 h (Figure 5C). Since the doubling time of HUVECs is slow (24 h), these results suggest that HLA-E is actively up-regulated in primary macrovascular ECs. In contrast, almost all EA.hy926 cells and HMECs initially showed a membrane HLA-E^+^ phenotype and contact with activated NK cells only enhanced the density of HLA-E expression (Figure 5C). In contrast to TKD/IL-2-activated NK cells, resting NK cells had no influence on the viability of the ECs, nor did they alter the expression of MIC-A, ICAM-1 (CD54) and HLA-E.

The dramatic up-regulation of ICAM-1 (CD54) and HLA-E on HUVECs was transient, since the levels of these proteins on the cell surface dropped to the very low baseline levels following the removal of activated NK cells (Figure 5D). Similar effects were seen with the increased density of these antigens on the immortalized EC types (data not shown).

### An Irradiation-Induced Up-Regulation of HLA-E Decreases Sensitivity to Activated, NKG2A and NKG2C Expressing NK Cells

**Figure 6:**
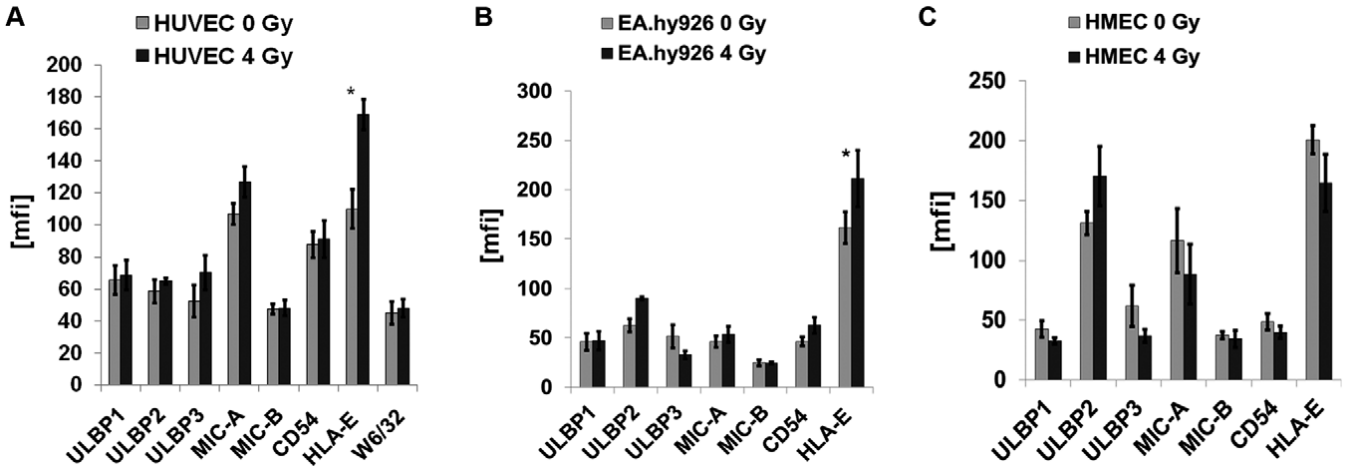
Phenotyping of ECs before and after irradiation at 4 Gy. Comparative analysis of the mean fluorescence intensity values for ULBP1-3, MIC-A/-B, CD54 (ICAM-1) and HLA-E expression on HUVECs (A), EA.hy926 cells (B) and HMECs (C) after ionizing irradiation at the sub-lethal dose of 4 Gy followed by a recovery period of 12 h. The MHC class I expression, as determined by W6/32 mAb, was measured only on HUVECs. The expression density of HLA-E was found to be significantly reduced on HUVECs and EA.hy926 cells (*, p<0.05), but not on HMECs following irradiation. No significant changes were observed with respect to other cell surface markers such as ULBP1-3, MIC-A/-B, CD54 and MHC class I antigens.

**Figure 7:**
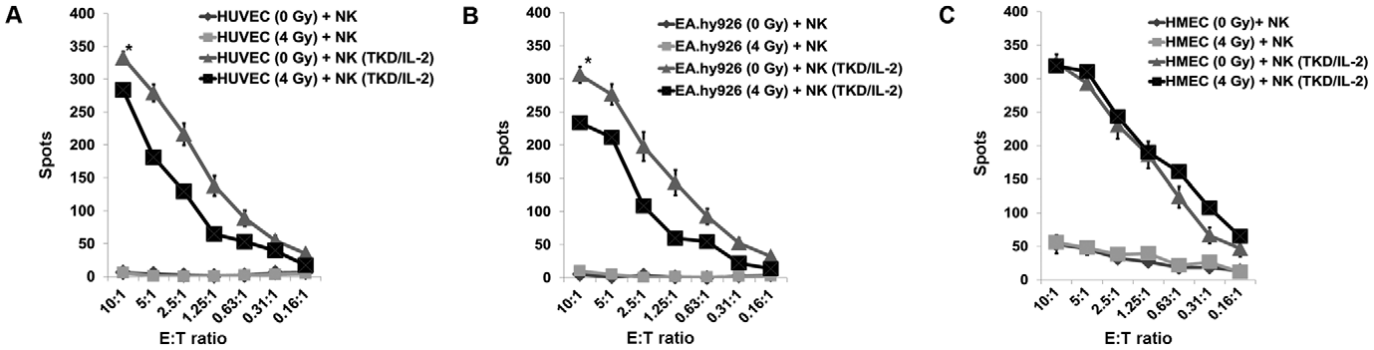
Comparative analysis of the lysis of non-irradiaited and irradiated ECs. Comparative analysis of the lysis of non-irradiated and irradiated (4 Gy) HUVECs (A), EA.hy926 cells (B) and HMECs (C) by un-stimulated and TKD/IL-2-stimulated NK cells. The data represent the mean values of three independent experiments and were obtained using 4 h granzyme B ELISPOT assays. The effector to target ratios ranged from 10∶1 to 0.16∶1. An asterisk marks values which are significantly different to values derived with non-irradiated target cells *, p<0.05 at all E∶T ratios.

Exposure to ionizing irradiation (4 Gy) followed by a recovery period of 12 h significantly increased the expression density of HLA-E on primary macrovascular HUVECs (Figure 6A) and also the percentage of HLA-E positive cells (from below 5 up to 27%). Similar effects were observed for the immortalized macrovascular partner cell line EA.hy926 (Figure 6B). Although these effects were similar to those induced by the contact of HUVECs with activated NK cells, they were less pronounced. In contrast, ionizing irradiation had no effect on the expression density of HLA-E on immortalized microvascular HMECs (Figure 6C). None of the other cell surface markers, such as MHC class I, ULBP1-3, MIC-A/-B and ICAM-1 (CD54), were found to be altered significantly following irradiation. In line with the increased cell surface expression of HLA-E, the lysis of irradiated HUVECs (Figure 7A) and EA.hy926 cells (Figure 7B) by activated NK cells expressing NKG2A and NKG2C receptors decreased significantly, whereas that of HMECs with an unaltered HLA-E membrane expression remained unaffected (Figure 7C). Un-stimulated NK cells did not affect the lysis of any of the EC types (Figure 7A–C) and also did not change the cell surface marker expression pattern as shown in Figure 5A–C.

**Figure 8:**
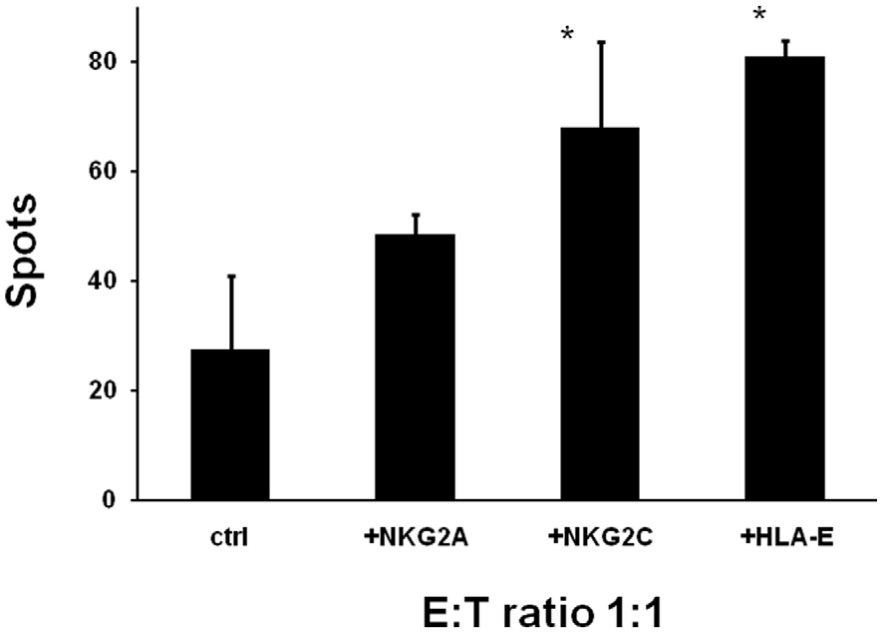
Blocking of lysis of irradiated ECs by antibodies directed against NK effector and target cells. Activation/inhibition of the granzyme B ELISPOT assay by antibodies directed against NKG2A, NKG2C or HLA-E. Comparative analysis of the lysis of irradiated (4 Gy) HUVECs either untreated or pre-incubated with HLA-E antibody by TKD/IL-2-stimulated NK cells that were pre-incubated either with NKG2A or NKG2C antibodies for 30 min. 50±3.5% expressed NKG2A and 68±15% of activated NK cells expressed NKG2C; 81±2.8% of irradiated HUVECs expressed HLA-E after contact with activated NK cells. The data represent the mean values of three experiments and were obtained using 4 h granzyme B ELISPOT assays. The effector to target (E∶T) ratio was 1∶1. An asterisk marks values which are significantly different to values derived with non-irradiated target cells *, p<0.05.

Pre-incubation of TKD/IL-2-activated NK cells with NKG2A and NKG2C antibodies and irradiated HUVECs (4Gy) with HLA-E antibodies, demonstrated that the lysis of irradiated HUVECs was significantly enhanced if NK cells had been pre-incubated with the agonistic NKG2C mAb (Figure 8). Furthermore, the blocking of the inhibitory ligand HLA-E on irradiated HUVECs using an HLA-E specific antibody results in a significantly enhanced lysis (Figure 8). In contrast, the lysis of irradiated HUVECs was not significantly affected by pre-incubating activated NK cells with the NKG2A mAb.

## Discussion

Herein, we have studied the expression of activating and inhibitory NK target ligands on human primary and immortalized ECs before and after irradiation and monitored its immunomodulatory functions. Although MHC class I, MHC class I related chain MIC-A and MIC-B and ULBP1-3 are frequently up-regulated on tumor cells by environmental stress [39], our findings indicate that a sub-lethal irradiation dose of 4 Gy does not significantly increase their cell surface expression on ECs. The MIC gene products [40] and ULBP1-3 proteins act as activating ligands for the C-type lectin receptor NKG2D [41]–[45] which is expressed on NK cells and via which activated NK cells can specifically kill their tumor target cells. We have previously shown that in contrast to normal tissues, tumors frequently present the major stress-inducible heat shock protein 70 (Hsp70) on their plasma membrane [37], [46]. Similar to MIC-A/-B and ULBP1-3 membrane Hsp70 also serves as recognition structure for NK cells that have been activated by the Hsp70 peptide TKD plus IL-2 (TKD/IL-2) [47], both on tumor cells *in vitro* and in tumor mouse models [48], [49]. Following activation, these NK cells show an elevated expression density of a panel of different receptors including the C-type lectin receptors CD94/NKG2A, CD94/NKG2C, NKG2D and natural cytotoxicity receptors (NCRs) [52], [53]. Furthermore, the Hsp70 membrane density on tumor cells can be selectively enhanced by ionizing irradiation. Here, we were interested to study the role of membrane Hsp70 as a recognition structure for NK cells on non-irradiated and irradiated ECs. As expected, only immortalized ECs exhibited a membrane Hsp70^+^ phenotype. We assume that the fusion of primary HUVECs with the membrane Hsp70^+^ tumor cell line A549-8, as well as the transformation of primary dermal microvascular ECs with the SV40 large T antigen, result in a malignant transformation of ECs which enables the translocation of Hsp70 to the plasma membrane in a manner which is analogous to that apparent for tumor cells. We therefore speculate that immortalized ECs might in part reflect the phenotype of a tumor cell. However, in contrast to tumor cells, neither primary and nor immortalized ECs exhibited significant up-regulation of membrane Hsp70 density after ionizing irradiation (data not shown).

Although HLA-E transcripts are always present in ECs [14], the HLA-E surface expression is highly variable. HLA-E is known to act as a negative regulatory signal for NK cells expressing the inhibitory receptor CD94/NKG2A and thus might confer immune regulatory functions [50], [51]. Trophoblasts and tumor cells protect themselves against the attack by the innate immune system via the expression of HLA-E [52], [53]. We have previously shown that transfection of HLA-E down-regulates the cytolytic response of TKD/IL-2-activated NK cells against tumor cells [54]. Along with these findings, HLA-E also plays a protective role against NK cells in the field of xenotransplantation. HLA-E transgenic pigs expressing human HLA-E on PBMNC and ECs were found to be protected against NK cell mediated killing [55]. When comparing the HLA-E phenotype in macro- and microvascular ECs with their susceptibility for NK cell mediated cytolysis, the absence of HLA-E was found to be associated with a higher sensitivity towards activated NK cells. In contrast, the expression of activating ligands, such as MIC-A/-B, ULBP1-3 and Hsp70 on ECs appears to be of minor importance for this NK cell mediated activity. A sub-lethal irradiation initiated a selective up-regulation of HLA-E on macrovascular ECs which correlated with a decreased susceptibility to NK cell mediated lysis. In line with these findings, treating of NK cells with an agonistic antibody directed against the activating NK cell receptor NKG2C and ECs with an antibody inhibiting HLA-E increased the lysis of irradiated, HLA-E expressing primary, macrovascular HUVECs. Since the lysis of irradiated HUVECs was not significantly affected by an antibody directed against the inhibitory receptor NKG2A, we speculate that the activating NKG2C receptor is dominant over the effects of the inhibitory receptor NKG2A.

Interestingly, HLA-E expression on immortalized microvascular ECs remained unchanged following irradiation and their lysis by activated NK cells remained unaltered. Although the physiological relevance of these differences in the HLA-E expression on macro- and microvasculature has yet to be elucidated, these findings might, among others, have future clinical implications for radiotherapy. Analyzing differences in activating and inhibiting NK ligands on ECs might be useful for therapeutic strategies that combine irradiation and NK cell-based therapies. Also it will be interesting to see the outcome of direct comparisons between normal ECs and ECs from malignant tumor samples of the same tissue which are currently being undertaken in our laboratory.

## Materials and Methods

### Cells

Human umbilical vein ECs (HUVEC) [34] are the most common source of primary macrovascular ECs used for in vitro studies. HUVECs were prepared by digestion using 0.1% collagenase/trypsin solution (Sigma-Aldrich Corp., St. Louis, MO, USA) and grown to confluence. The cells were cultured in T25 flasks at a density of 1×10^6^ cells per ml in ECGM medium supplemented with 10% v/v FCS and Supplement Mix containing 100 IU/ml polymyxin B in an incubator at 37°C in a humidified atmosphere with 5% CO_2_. Cells were passaged twice a week and harvested by incubation with trypsin/EDTA for 1 min at 37°C. Cells undergo senescence at passages 8–10.

The cell line EA.hy926 results from a fusion of HUVECs with the epithelial lung tumor cell line A549-8 [56]. The cell line EA.hy926 was kindly provided by Dr. Cora-Jean S. Edgell (University of North Carolina, Chapel Hill, USA). The cells were grown in DMEM medium supplemented with 10% v/v FCS, 2 µM glutamine, 100 µM hypoxanthine, 0.4 µM aminopterin, 16 µM thymidine and 50 mg/l gentamicin at 37°C in a humidified atmosphere with 5% CO_2_.

The human microvascular endothelial cell line CDC/EU.HMEC-1, further referred as HMEC, was derived by transfecting human dermal microvascular ECs using a PBR-322 plasmid containing the coding region for the simian virus 40 A gene product (SV40), large T antigen to immortalize them [57]. The cell line was cultured in MCDB 131 medium (Gibco BRL, Karlsruhe, Germany) supplemented with 15% v/v FCS, 2 µM glutamine (Gibco BRL), 100 µM hydrocortisone (Sigma Aldrich, Deisenhofen, Germany), 10 ng/ml Epidermal growth factor (EGF, Becton Dickinson, Heidelberg, Germany) and 1% w/v penicillin/streptomycin (Gibco BRL, Karlsruhe, Germany) as antibiotics at 37°C in a humidified atmosphere with 5% CO_2_.

### Irradiation of ECs

Macro- and microvascular primary and immortalized ECs (HUVECs, EA.hy926 cells and HMECs) were cultured to 75% confluence and then irradiated with a single dose of 4 Gy at a dose rate of 1 Gy/min (Gulmay Isodose Control, Solingen, Germany). After a recovery period of 12 h, supernatants were centrifuged to remove the detached cells. The surviving cell fraction was used for further analysis. The total number of the attached and detached cells was counted.

### Von Willebrand Factor Staining

For the von Willebrand (factor VIII-related antigen) staining, cells were seeded into 8-well µ-slide. After adherence, the cells were fixed with methanol-acetone (1∶1) at room temperature for 2 min and rinsed with PBS. Samples were incubated with polyclonal rabbit antibody against human factor VIII-related antigen (Sigma Aldrich, Deisenhofen, Germany) at 37°C for 45 min, rinsed with PBS and incubated with goat-anti-rabbit FITC (fluorescein-isothiocyanate)-labeled secondary antibody (Sigma Aldrich, Deisenhofen, Germany) at 37°C for another 30 min. Cells were then washed with PBS/10% v/v FCS and cell nuclei were stained with 1 µg/ml diamidinophenylindole (DAPI, Roche, Mannheim, Germany).

### Flow Cytometry

Cells were detached from the culture flasks using trypsin/EDTA (Gibco BRL, Karlsruhe, Germany) at 37°C for 1 min. After two washings in ice-cold PBS/FCS (1% v/v) cells were incubated for 30 min at 4°C with the following fluorescence-conjugated monoclonal antibodies (mAbs): anti-CD31 (Becton Dickinson, Heidelberg, Germany), anti-ULBP1-3, anti-MICA/B (BAMO1, IgG1; BAMO2, IgG2a, Bamomab, Munich, Germany), anti-HLA-E (MEM-06, Biozol, Eching, Germany), anti-MHC class I (W6/32, Sigma, Missouri, USA), anti-Hsp70 (cmHsp70.1, multimmune GmbH, Munich, Germany; SPA810, Stressgen via Assay Designs, Ann Arbor, MI, USA), anti-CD54 (Dianova, Hamburg, Germany). After another two washing steps viable, propidium iodide-negative cells were gated and analyzed on a FACSCalibur flow cytometer (Becton Dickinson, Heidelberg, Germany).

### ^51^Cr Release and Granzyme B ELISPOT Assays and Inhibition/Activation studies

Briefly, viable ECs were labeled with 0.1 µCi of Na_2_^51^CrO_4_ (Hartmann Analytic GmbH, Braunschweig, Germany) at 37°C for 2 h. After two washes with RPMI 1640 medium, ^51^Cr-labeled target cells (1×10^4^) were transferred into triplicate wells of a 96-well plate. Human NK cells which were isolated using a standard CD3/CD19 depletion protocol (Miltenyi, Dreieich, Germany), followed by an adherence selection of CD14^+^ cells. Un-stimulated NK cells or NK cells stimulated with TKD (2 µg/ml) and IL-2 (100 IU/ml) were then added to the ECs at various effector to target (E∶T) cell ratios. After a 4 h co-incubation, supernatants (100 µl) were harvested and their radioactivity determined was using a gamma counter (Coulter). Specific lysis was calculated using the formula: % specific lysis  =  (experimental release – spontaneous release)/(maximum release –spontaneous release) ×100. The spontaneous release for each target cell ranged between 10 and 15%.

For the granzyme B ELISPOT assay, 96-well ELISPOT plates (Millipore GmbH, Schwalbach, Germany) were coated with capture antibody by an overnight incubation at 4°C, after which they were blocked using 10% v/v FCS. The effector and target cells (3×10^3^) were added at different E∶T ratios as indicated. After 4 h incubation at 37°C and 2 washing steps, a biotinylated detecting antibody (2 µg/ml) was added. After additional 2 washes, the presence of granzyme B was visualized using 3-amino-9-ethyl-carbazole substrate solution (25 min). Spots were counted and data were analyzed using an Immuno Spot Series 3A Analyzer (CTL-Europe GmbH, Aalen, Germany).

For inhibition/activation studies effector (TKD/IL-2-activated NK cells) or target (HUVECs) cells were pre-incubated with unlabeled NKG2A, NKG2C (MAB1059, MAB1381, 5 µg/ml each, R&D systems, Minneapolis, MN, USA) or HLA-E (MEM-06, Biozol, Eching, Germany, 5 µg/ml) antibodies for 30 min at room temperature and then used in the granzyme B ELISPOT assay at an E∶T ratio of 1∶1.

### Statistical Analysis

Means between two groups were tested for differences using the t-test or the non-parametric Mann Whitney rank sum test, means of more than two groups were compared using the Analysis of variance (ANOVA).

### IBIDI Movie

Due to the better availability of EC lines compared to primary ECs, HMECs were used as target cells for the videos. Killing of HMECs by resting and TKD/IL-2-activated human NK cells at an effector to target (E∶T) ratio of 5∶1. Briefly, 5,000 HMEC were seeded into µ Dishes ^35 mm, low^ with Culture Insert (IBIDI, Martinsried; Germany) in a volume of 70 µl RPMI-1640 medium supplemented with 10% v/v FCS and cultured overnight. The supernatant was removed and 25,000 NK cells (resting or IL-2 activated for 4 days) were added in a volume of 70 µl fresh medium. Killing of target cells was filmed for 6 h. Time interval, 20 s; number of slides, 1200; totally elapsed 6 h, magnification, 10×, MV; format, mpg.

## Supporting Information

Video S1 **Movie illustrating the attack of HMECs by resting NK cells.** Resting NK cells co-cultured with HMECs at an E:T ratio of 10:1 for 6 h at a magnification of 10x.
(MP4)

Video S2 **Movie illustrating the attack of HMECs by TKD/IL-2-activated NK cells.** TKD/IL-2-activated NK cells co-cultured with HMECs at an E:T ratio of 10:1 for 6 h at a magnification of 10x.
(MP4)

Video S3 **Movie illustrating the attack of HMECs by TKD/IL-2-activated NK cells.** TKD/IL-2-activated NK cells co-cultured with HMECs at an E:T ratio of 10:1 for 6 h at a magnification of 20x.
(MP4)
